# Optical Coherence Tomography Angiography in *CRB1*-Associated Retinal Dystrophies

**DOI:** 10.3390/jcm12031095

**Published:** 2023-01-31

**Authors:** Firuzeh Rajabian, Alessandro Arrigo, Lorenzo Bianco, Alessio Antropoli, Maria Pia Manitto, Elisabetta Martina, Francesco Bandello, Jay Chhablani, Maurizio Battaglia Parodi

**Affiliations:** 1 Department of Ophthalmology, Vita-Salute San Raffaele University, IRCCS Ospedale San Raffaele, 20132 Milan, Italy; 2 Department of Ophthalmology, University of Pittsburgh School of Medicine, Pittsburgh, PA 15213, USA

**Keywords:** *CRB1*, crumbs homolog 1, retinal dystrophy, optical coherence tomography angiography, OCTA, multimodal imaging

## Abstract

Aim of the study: To report optical coherence tomography angiography (OCTA) findings in patients affected by *CRB1*-associated retinal dystrophies. Method: Patients affected by a genetically confirmed *CRB1*-associated retinal dystrophy were prospectively enrolled in an observational study, along with age- and sex-matched healthy volunteers as control subjects. All study and control subjects received a complete ophthalmic examination and multimodal retinal imaging, including OCTA. Result: A total of 12 eyes from 6 patients were included in the study. The mean BCVA of patients was 0.42 ± 0.25 logMAR. Two patients showed large central atrophy, with corresponding definite hypo-autofluorescence on fundus autofluorescence (FAF). Another four patients disclosed different degrees of RPE mottling, with uneven FAF. On OCTA, the macular deep capillary plexus and choriocapillaris had a lower vessel density in eyes affected by *CRB1*-associated retinopathy when compared to healthy controls. On the other hand, vessel density at the peripapillary radial capillary plexus, superficial capillary plexus, and deep capillary plexus was significantly altered with respect to control eyes. Statistical analyses disclosed a negative correlation between the deep capillary plexus and both LogMAR best corrected visual acuity and central retinal thickness. Conclusion: Our study reveals that *CRB1*-associated retinal dystrophies are characterized by vascular alterations both in the macular and peripapillary region, as assessed by OCTA.

## 1. Introduction

*CRB1*-associated retinal dystrophy is a rare inherited disease (IRD) characterized by variable phenotypic manifestations, ranging from retinitis pigmentosa and Leber congenital amaurosis to isolated macular dystrophies [1,2,3,4,5]. While a recent study suggested that the more severe and early-onset forms of retinal degeneration are associated with null variants [6], a previous meta-analysis suggested that the different phenotype of patients with *CRB1* variants is possibly influenced by additional modifying factors rather than being determined by specific allelic combinations [7]. The degree of visual impairment is highly dependent on the specific phenotype—low vision in individuals with Leber congenital amaurosis and retinitis pigmentosa is reached in the second and fourth decade of life, respectively [8], while patients with macular dystrophy retain a relatively good visual function until adult age in at least one eye [6]. Optical coherence tomography (OCT) features of *CRB1*-associated retinal dystrophies have been extensively described and include abnormal retinal lamination, macular cystoid changes, and increased retinal nerve fiber layer thickness, and [6,9,10,11]. 

Optical coherence tomography angiography (OCTA) has been used in several IRDs to characterize the vascular anatomy in the macula and to identify vascular patterns associated with a faster progression. In particular, a reduction in vessel density at the level of the deep capillary plexus (DCP) has been described in several IRDs, including Stargardt disease [12], cone dystrophies [13], Best vitelliform macular dystrophy [14], X-linked retinoschisis [15], choroideremia [16], occult macular dystrophy [17], congenital stationary night-blindness [18], retinitis pigmentosa [19,20], and Bietti crystalline dystrophy [21]. This study aimed to describe the OCTA features in eyes affected by *CRB1*-associated retinal dystrophies, as no prior study has explored the vascular alterations in these disorders.

## 2. Methods

This cross-sectional case series included patients affected by an IRD related to a mono- or biallelic *CRB1* variant, detected by means of next-generation sequencing. A group of healthy age- and sex-matched control subjects was also enrolled. The study adhered to the tenets of the Declaration of Helsinki and was approved by the Institutional Review Board (MIRD2020) of IRCCS San Raffaele Hospital. Written informed consent was obtained from all the subjects included in the study. 

The patients underwent an ophthalmological examination, complete with best corrected visual acuity (BCVA) measurement using standard ETDRS charts, slit-lamp examination, and multimodal retinal imaging. The standard imaging protocol included color photography, spectral-domain optical coherence tomography (OCT), and blue-light autofluorescence (FAF) (Spectralis HRA+OCT, Heidelberg Engineering, Heidelberg, Germany). Optical coherence tomography angiography (OCTA) (SS-DRI OCT Triton, Topcon, Tokyo, Japan) scans were of 4.5 × 4.5 mm volumes, acquired both in the macula and optic nerve head. Only high-quality images, assessed by Topcon image quality index (≥70) [2], were considered.

In order to obtain macular and peripapillary vessel density (VD) measures, automatic segmentation of all vascular plexuses was first obtained from native OCTA acquisitions on ImageNet6 software; segmentations were manually corrected by an expert ophthalmologist (FR). Reconstructions of the superficial capillary plexus (SCP), deep capillary plexus (DCP), and choriocapillaris (CC), as well as the radial peripapillary capillary (RPC) plexus in optic nerve head scans, were then exported from the instrument in the .tiff format and imported into ImageJ software Version 1.53h (National Institutes of Health, Bethesda, MD, USA). All images were binarized using a mean threshold, to reduce the noise and highlight the blood vessels. Then, the white region was considered as the vascular area, and its number of pixels was quantified and expressed as a percentage over the total after exclusion of the foveal avascular zone (i.e., the VD parameter). Macular parameters will be referred to by means of the prefix “m”, whereas the ones related to the optic nerve head with “n” (e.g., mSCP and nSCP for macular and peripapillary SCP, respectively) [22].

An unpaired two-tailed t-test (SPSS; Chicago, IL, USA) was used to compare the quantitative parameters among affected and control eyes. Correlations were assessed by means of the Pearson correlation coefficient. Statistical significance was set at *p* ≤ 0.05.

## 3. Results

Overall, a total of 6 patients (12 eyes) affected by a genetically confirmed *CRB1*-associated retinal dystrophy were recruited, with ages ranging between 10 and 67 years (mean age 36.4 ± 25.7 years) and a mean BCVA of 0.4 ± 0.25 logMAR (Table 1). The control group consisted of six age- and sex-matched healthy volunteers.

Anterior segment examination revealed no alteration in all patients. Two patients showed a large central atrophy, with corresponding definite hypo-autofluorescence on FAF. Another four patients disclosed different degrees of RPE mottling, with uneven FAF response. In all cases lesions turned out to be symmetrical between the two eyes of the same patient.

**Figure 1:**
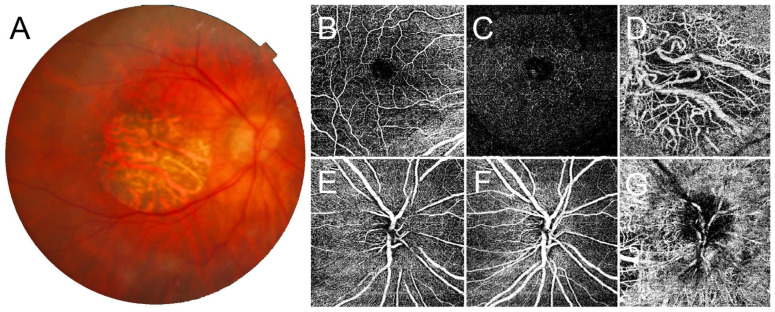
Optical coherence tomography angiography (OCTA) findings in *CRB1*-associated retinal dystrophy presenting with macular atrophy. Color fundus photography (**A**) shows complete atrophy of the macular region. Here, OCTA detects a partially spared superficial capillary plexus (**B**), markedly disrupted deep capillary plexus (**C**), and completely absent choriocapillaris (**D**) in the macula. At the level of the optic nerve head, it is possible to observe a loss of the radial peripapillary capillary plexus (**E**), together with a rarefied superficial and deep capillary plexus (**F**), while the choriocapillaris appears preserved (**G**).

**Figure 2:**
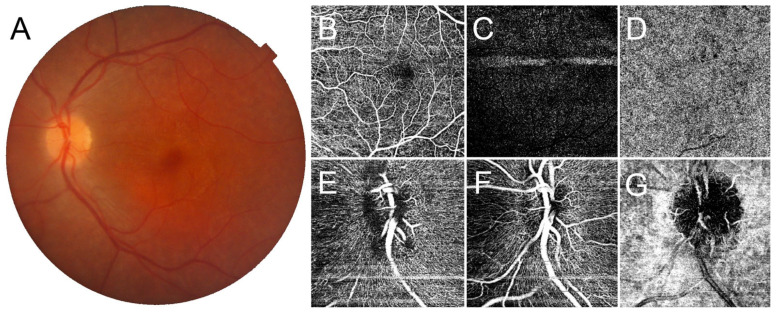
Optical coherence tomography angiography (OCTA) findings in *CRB1*-associated retinal dystrophy presenting with macular mottling. Color fundus photography (**A**) is characterized by macular pigmentary alterations in absence of clear atrophy. In the macula, OCTA detects a spared superficial capillary plexus (**B**), markedly disrupted deep capillary plexus (**C**), and choriocapillaris flow voids (**D**). At the level of the optic nerve head, the radial peripapillary capillary plexus appears rarefied (**E**), together with the involvement of the superficial and deep capillary plexus (**F**), while the choriocapillaris appears preserved (**G**).

Central retinal thickness (CRT) was lower in *CRB1* patients compared to control eyes (164 ± 56.7 vs. 256 + 45 µm), while subfoveal choroidal thickness (SFCT) was similar (250 ± 95 vs. 251 ± 118 µm). Considering macular vascular plexa, OCTA detected an almost preserved VD at SCP (*p* > 0.05) while this was significantly reduced at DCP and CC (*p* < 0.05). At the level of the optic nerve head, VD at RCP, SCP, and DCP were significantly lower than control eyes (*p* < 0.05) (Table 2). Significant negative correlations were found between VD at macular DCP and both LogMAR BCVA (*r* = −0.71; *p* < 0.001) and CRT (*r* = −0.62; *p* < 0.001). The OCTA imaging in two cases of *CRB1*-associated retinal dystrophy is reported in Figure 1 and Figure 2. 

## 4. Discussion

In the present study, we describe OCTA findings in six patients affected by genetically confirmed *CRB1*-associated retinal dystrophy. The OCTA examination indicates that this IRD is characterized by an extensive rarefaction of intraretinal vasculature and CC. In more detail, we detected a VD reduction at the level of DCP and CC in the macula, and at the level of RCP, SCP, and DCP in the peripapillary region. Our data are based on simple cross-sectional analyses, making it difficult to tell whether the vascular impairment is primary feature or, instead, a secondary phenomenon due to degenerative changes in the photoreceptor-RPE and inner retinal layers. However, it should be noted that *CRB1* variants been described in association with Coats-like exudative vasculopathy both in Leber congenital amaurosis and retinitis pigmentosa [23,24,25].

The CRB1 (Crumbs homologue 1) protein belongs to the CRB complex, which functions in the maintenance of apical–basal cell polarity and the formation of adherent junctions between cells [26]. As the *CRB1* gene in the human retina is expressed in Muller glial microvilli and photoreceptor cells but is absent in retinal pigment epithelium and choroid tissue [27,28], we believe that the vascular rarefaction detected within the macular region likely represents a secondary effect. However, significant rarefaction of intraretinal vasculature was also identified in the peripapillary region, and the interpretation of this latter finding may be related to a diffuse dysregulation of the retinal neurovascular unit, which is composed of neuronal cells, intraretinal vessels, and *CRB1*-expressing Muller cells [29]. Interestingly, the severity of the vessel density reduction was similar across the different phenotypical manifestations, which ranged from mere RPE mottling up to large atrophic changes, suggesting that this peripapillary vascular impairment is independent of the stage of the disease. Even though the comparison of the OCTA findings described in other IRDs is hard due to the different pathogenesis of each subform, we have to underline that DCP is always involved, often also showing a correlation with BCVA [12,13,14,15,16,17,18,19,20,21]. Thus, DCP may represent an important biomarker to better characterize the stage of the disease and the extent of functional damage.

We are aware that our case series has a number of limitations, including, first of all, the scant number of patients and the inclusion of both eyes in the analysis. Nevertheless, *CRB1*-associated retinal dystrophy is a rare condition. Moreover, the different phenotypic manifestations and the different stages could be characterized by variable vascular alterations. Multicenter studies with a longitudinal follow-up should be designed to collect a high number of patients affected by *CRB1*-associated retinal dystrophy to ascertain the natural history evolution, investigating the correlation between dystrophy severity and vascular damage. In addition, quantitative analysis of OCTA images in the setting of IRDs is hindered by the inherent difficulty of segmenting individual vascular plexuses, especially in the presence of extensive atrophy or severely altered retinal lamination [22]. Lastly, we cannot exclude that the phenotype and pattern of vascular impairment that we observed our cohort could have been modified by allelic variants in other genes related to IRDs.

In essence, our study highlights that *CRB1*-associated retinal dystrophy is characterized by vascular alterations on OCTA both in the macula and peripapillary region. Further investigations are warranted to confirm our results and the relationship between the severity of clinical phenotype and the extent of vascular damage. This knowledge could eventually provide new insights on the pathogenesis of this IRD, as well as useful criteria for patient selection in future gene therapy trials.

## Figures and Tables

**Table 1:** Clinical and imaging characteristics of patients affected by *CRB1*-associated retinal dystrophies.

	Gender	BCVA (logMAR)	CRT (μm)	SFCT (μm)	*CRB1* Variants
Patient 1	M	0.4	173	465	c.772_779delinsG; c.498_506del
		0.4	163	340	
Patient 2	M	0	223	274	c.498_506del
		0.3	210	285	
Patient 3	M	0.2	220	239	c.1584C>A; c.498_506del
		0.3	158	251	
Patient 4	F	0.2	189	74	c.614T>C
		0.7	29	56	
Patient 5	M	0.7	151	290	c.2549G>T; c.4176_4177delAA
		0.7	131	238	
Patient 6	F	0.4	178	276	c.614T>C
		0.8	110	280	

Legend—For each patient, the first row refers to the right eye. Abbreviations—best corrected visual acuity (BCVA), central retinal thickness (CRT), subfoveal choroidal thickness (SFCT).

**Table 2:** Vessel density analysis at individual vascular plexa in *CRB1*-associated retinal dystrophy and control eyes.

	mSCP	mDCP	mCC	nRCP	nSCP	nDCP	nCC
CRB1	0.405 ± 0.013	0.360 ± 0.031	0.482 ± 0.014	0.398 ± 0.024	0.395 ± 0.037	0.305 ± 0.038	0.513 ± 0.042
Controls	0.413 ± 0.012	0.434 ± 0.005	0.500 ± 0.006	0.443 ± 0.007	0.426 ± 0.010	0.402 ± 0.019	0.542 ± 0.031
*p*-value	0.13352	4.46 × 10^−9^ *	0.000138 *	4.71 × 10^−7^ *	0.005277 *	1.70 × 10^−8^ *	0.060817

Legend—Data are presented as mean value and SD; * = *p*-value < 0.05; macular superficial capillary plexus (mSCP), macular deep capillary plexus (mDCP), macular choriocapillaris (mCC), nerve radial capillary plexus (nRCP), nerve superficial capillary plexus (nSCP), nerve deep capillary plexus (nDCP), nerve choriocapillaris (nCC).

## Data Availability

The data presented in this study are available on request from the corresponding author. The data are not publicly available due to ethical restrictions.
